# Demographic transition and factors associated with remaining in place after the 2011 Fukushima nuclear disaster and related evacuation orders

**DOI:** 10.1371/journal.pone.0194134

**Published:** 2018-03-14

**Authors:** Tomohiro Morita, Shuhei Nomura, Tomoyuki Furutani, Claire Leppold, Masaharu Tsubokura, Akihiko Ozaki, Sae Ochi, Masahiro Kami, Shigeaki Kato, Tomoyoshi Oikawa

**Affiliations:** 1 Department of Research, Minamisoma Municipal General Hospital, Minamisoma City, Fukushima, Japan; 2 Internal Medicine, Soma Central Hospital, Soma City, Fukushima, Japan; 3 Division of Social Communication System for Advanced Clinical Research, Institute of Medical Science, The University of Tokyo, Minato-ku, Tokyo, Japan; 4 Department of Global Health Policy, Graduate School of Medicine, The University of Tokyo, Bunkyo-ku, Tokyo, Japan; 5 Department of Epidemiology and Biostatistics, School of Public Health, Imperial College London, Norfolk Place, London, United Kingdom; 6 Faculty of Policy Management, Keio University, Fujisawa, Kanagawa, Japan; 7 Global Public Health Unit, School of Social and Political Science, University of Edinburgh, George Square, Edinburgh, United Kingdom; 8 Medical Governance Research Institute, Minato-ku, Tokyo, Japan; 9 Research Institute of Innovative Medicine, Tokiwa Foundation, Iwaki, Fukushima, Japan; 10 Center for Regional Cooperation Iwaki Meisei University, Iwaki, Fukushima, Japan; Senior Research Associate, Pentland Centre for Sustainability in Business, Lancaster University Management School, Lancaster, UNITED KINGDOM

## Abstract

**Introduction:**

Demographic changes as a result of evacuation in the acute phase of the 2011 Fukushima nuclear disaster are not well evaluated. We estimated post-disaster demographic transitions in Minamisoma City—located 14–38 km north of the nuclear plant—in the first month of the disaster; and identified demographic factors associated with the population remaining in the affected areas.

**Materials and methods:**

We extracted data from the evacuation behavior survey administered to participants in the city between July 11, 2011 and April 30, 2013. Using mathematical models, we estimated the total population in the city after the disaster according to sex, age group, and administrative divisions of the city. To investigate factors associated with the population remaining in place after the disaster, a probit regression model was employed, taking into account sex, age, pre-disaster dwelling area, and household composition.

**Results:**

The overall population decline in Minamisoma City peaked 11 days after the disaster, when the population reached 7,107 people—11% of the pre-disaster level. The remaining population levels differed by area: 1.1% for mandatory evacuation zone, 12.5% for indoor sheltering zone, and 12.6% for other areas of the city. Based on multiple regression analyses, higher odds for remaining in place were observed among men (odds ratio 1.72 [95% confidence intervals 1.64–1.85]) than women; among people aged 40–64 years (1.40 [1.24–1.58]) than those aged 75 years or older; and among those living with the elderly, aged 70 years or older (1.18 [1.09–1.27]) or those living alone (1.71 [1.50–1.94]) than among those who were not.

**Discussion:**

Despite the evacuation order, some residents of mandatory evacuation zones remained in place, signaling the need for preparation to respond to their post-disaster needs. Indoor sheltering instructions may have accelerated voluntary evacuation, and this demonstrates the need for preventing potentially disorganized evacuation in future nuclear events.

## Introduction

Natural and manmade disasters, such as earthquakes, floods, and fires often lead to mass evacuation and/or relocation [1]. In 2015, 27.8 million people worldwide were newly displaced by evacuation or relocation due to disasters [2]. Among different types of disasters, nuclear/radiation-related disasters pose particularly unique challenges in the context of evacuation, since fear, anxiety and stress regarding exposure to radiation—an invisible threat—act to magnify the scale and scope of the evacuation [3]. In the case of the 1987 Chernobyl nuclear disaster, more than 350 thousand people living within several hundred kilometers from the nuclear plant were evacuated because of radiation-specific reasons [4, 5]. Countermeasures in the wake of nuclear disasters include rapid (and often unplanned) evacuation to avoid acute radiation-related health consequences; however these countermeasures may also exert a powerful, direct health burden on the evacuees' well-being, and may potentially lead to an alarming rise in morbidity and mortality [6]. Importantly, mass evacuations can also have adverse effects on the functioning of health systems in local communities or societies in affected areas (e.g. disruptions in the delivery of healthcare and other public health services), which in turn may expose those who remain in place to new health risks.

The design and delivery of measures to manage post-disaster health risks and improve health outcomes can be achieved by understanding population demographics, particularly in the acute phase of the disaster, when immediate decisions about use of protective countermeasures are urgently required to be taken [7]. In the case of nuclear disasters, radiation monitoring systems have been used to support evacuation decision making, such as Real-time On-line Decision Support (RODOS) in Europe [8] or the System for Prediction of Environment Emergency Dose Information (SPEEDI) in Japan [9]. Since different demographics engender differential exposure to post-disaster health-compromising conditions (e.g. living and working conditions, housing, access to healthcare and education), they require different policy responses [10]. However, to our knowledge, lessons on how local communities undergo demographic transitions in mass evacuations following nuclear disasters are not well-documented, and thus may not provide sufficient knowledge to allow for informed policy responses.

**Fig 1:**
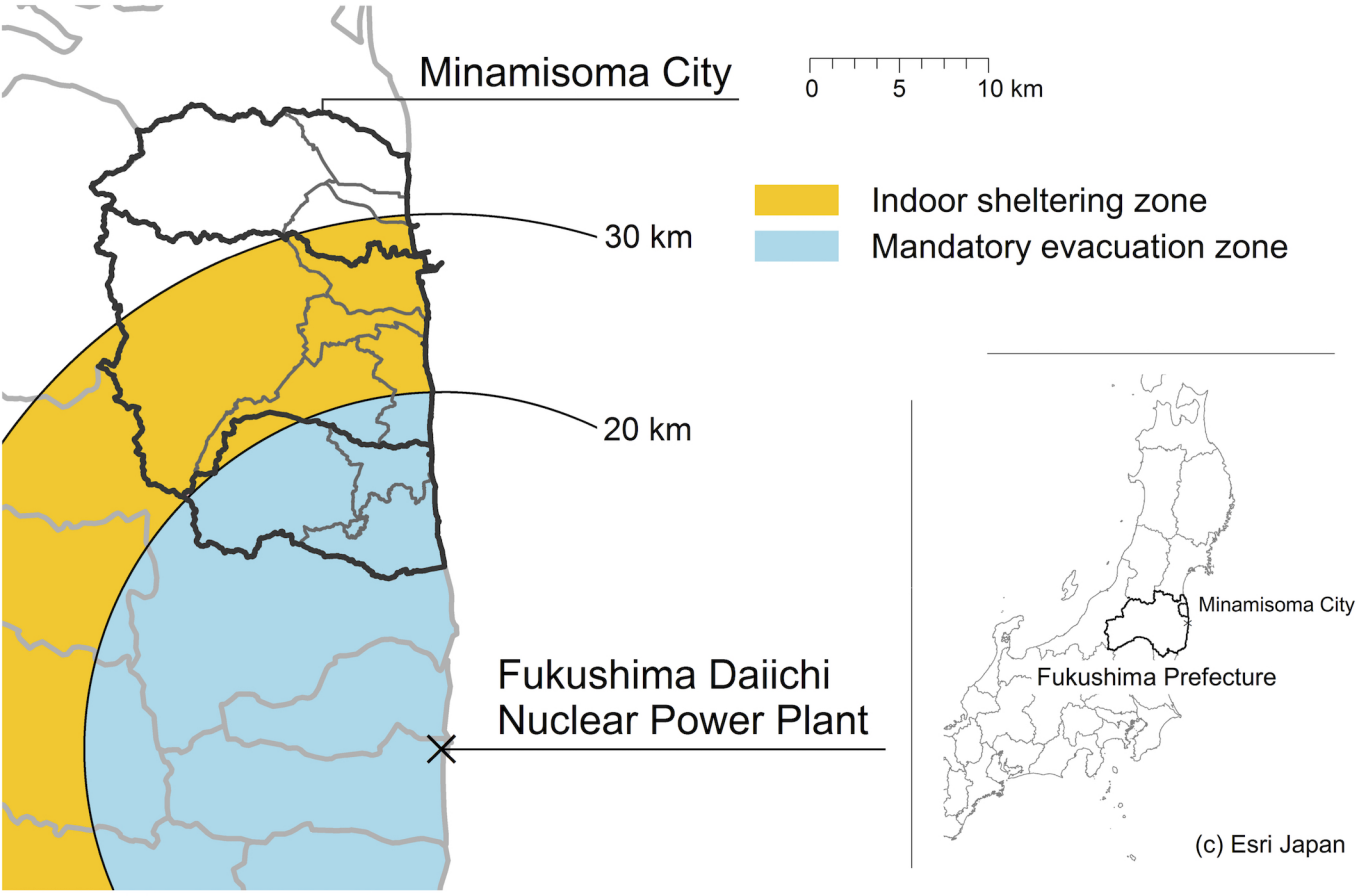
Location of Minamisoma City.

Mass evacuation is exemplified in the case of the 2011 Fukushima Daiichi nuclear power plant disaster, which followed the Great East Japan Earthquake and Tsunami on March 11, 2011. Following a series of government evacuation orders, including mandatory evacuation orders, within a 20 km radius of the power plant, and indoor sheltering instructions, issued within a 20–30 km radius of the power plant, over 160 thousand people from areas surrounding the nuclear power plant relocated within Fukushima Prefecture, or moved out of the Prefecture, with some people moving hundreds of kilometers away from the plant, and some moving several times [11]. Minamisoma City, located 14–38 km north of the Fukushima nuclear plant (Fig 1), is a unique city, in that residents were subjected to a range of evacuation instructions and countermeasures, and likely experienced anxiety and fear of radioactive exposure after the disaster. Consequentially, there was a substantial population loss, from the pre-disaster population of nearly 72,000, to a post-disaster population of approximately 10,000 within the first month after the disaster [12, 13]. This city can therefore be seen as a uniquely strong candidate for evaluation of how mass evacuation can affect the demographics of remaining residents.

Minamisoma City is also the first local authority that initiated an internal radiation contamination-screening program for residents after the Fukushima disaster [14]. As part of this screening, the city conducted a questionnaire survey to collect detailed information on the post-disaster evacuation behavior of the participants, including the location and time-period of their evacuation(s). This survey enabled us to (1) estimate the post-disaster demographic transitions in Minamisoma City in the first month after the disaster (March 11 to March 31, 2011); and (2) identify the potential demographic factors associated with the population remaining in the affected areas. The aim of this study was to enhance the understanding of changes in population demographics after major nuclear disasters; this was done with the goal of identifying entry points for action, and developing strategic directions for health policy and practice during acute phase disaster risk management.

## Materials and methods

### Settings

#### Study site–Minamisoma City

On March 12, 2011, the central government declared a nuclear emergency in response to the Fukushima nuclear disaster, and ordered evacuation of all areas within a 20 km radius of the Fukushima Daiichi nuclear power plant (mandatory evacuation zone) or with high ambient radiation dose; on March 15, residents within a 20–30 km radius of the power plant were instructed to stay indoors (indoor sheltering zone). Minamisoma City includes areas that fall into both the mandatory evacuation and indoor sheltering zones, where about 10,955s and 44,773 people originally lived, representing 16% and 66% of the total population of the city, respectively. The geographical range that falls under government evacuation orders is shown in Fig 1.

#### Whole Body Counter screening program

In response to the disaster, Minamisoma City launched an internal radiation exposure screening program for the city residents in July 2011, using whole body counter (WBC) units—two chair-type units (Anzai Medical Co., LTD, Japan and Fuji Electric Co., LTD, Japan), which were replaced with standing-type units (FASTSCAN Model 2251, Canberra Inc., United States) in September 2011. The WBC units were installed at Minamisoma Municipal General Hospital (MMGH), located 23 km north of the Fukushima nuclear plant, and still remain at this location. The screening program was provided to the residents free of charge. Only residents aged 6 years or older were eligible to be screened, because the WBC units were not suitable for measurement in small children (i.e. in pre-school children who are younger than 6 years old) [15]. A program notification was sent to each household, including former residents who had evacuated to other areas, but could be tracked using the city's family registry. The program notification was also circulated using the hospital website, and the city's public relations magazine [14].

A total of 20,149 individuals participated in the WBC screening program at MMGH during the study period (July 11, 2011 to April 30, 2013), and answered the questionnaire survey on post-disaster evacuation behavior. The sex and age of the participants in the first round of screening, as well as their household information at the time of the WBC screening, are shown in Table 1. With respect to the pre-disaster dwelling area, more than half of the WBC participants lived in the indoor sheltering zone before the disaster (13,801, [68%]), while 3,415 (17%) lived in the mandatory evacuation zone and 2,933 (15%) lived in other areas of the city. Fifty-five percent (11,032/20,149) of the participants were female. Mean and standard deviation (SD) of the participants’ age were 41 and 23. Nineteen percent (3,752/20,149) of the participants were aged more than 65 years old. As for households, the percentages of those living with pre-school children, and those living with an elderly person aged 70 years or older were 14% and 28%, respectively, while a few participants lived alone (3%). Among the WBC participants, those who evacuated, either on mandatory orders or voluntarily, to areas outside Minamisoma City (evacuees) before March 31, 2011 from the mandatory evacuation zone, indoor sheltering zone, and other areas of the city, were 3,394 (99%), 12,771 (93%), and 2,697 (92%), respectively.

**Table 1:** Demographic characteristics of the WBC participants (n = 20,149) by dwelling area from July 11, 2011 to April 30, 2013.

Pre-disaster dwelling area (n)	Mandatory evacuation zone 3,415	Indoor sheltering zone 13,801	Other areas in the city 2,933	Total (whole areas) 20,149
Sex (n, %)				
Male	1,526 (45)	6,250 (45)	1,341 (46)	9,117 (45)
Female	1,889 (55)	7,551 (55)	1,592 (54)	11,032 (55)
Age at WBC screening [years]				
Mean (SD)	42 (22)	42 (23)	38 (23)	41 (23)
By group (n, %)				
6–9	228 (7)	981 (7)	279 (10)	1488 (7)
10–14	331 (10)	1633 (12)	484 (17)	2448 (12)
15–19	327 (10)	1182 (9)	214 (7)	1723 (9)
20–39	659 (19)	2550 (18)	573 (20)	3782 (19)
40–64	1302 (38)	4736 (34)	918 (31)	6956 (35)
65–74	337 (10)	1732 (13)	295 (10)	2364 (12)
75–	231 (7)	987 (7)	170 (6)	1388 (7)
Household at WBC screening (n, %)				
Living with pre-school children	541 (16)	1,730 (13)	524 (18)	2,795 (14)
Living with an elderly person aged 70 years or older	1,133 (33)	3,617 (26)	884 (30)	5,634 (28)
Living alone	65 (2)	565 (4)	55 (2)	685 (3)
Evacuation behaviors (n, %)*				
Evacuees	3,394 (99)	12,771 (93)	2,697 (92)	18,862 (94)
Remainees	21 (1)	1,030 (7)	236 (8)	1,287 (6)

SD: standard deviation.

*Those who evacuated to areas outside Minamisoma City once or more before March 31, 2011 were defined as ‘evacuees’ (even if they returned to the city or to the original pre-disaster dwelling area before this date); and others as ‘remainees’.

#### Evacuation behavior survey among WBC participants

Minamisoma City conducted a questionnaire survey for all the WBC screening participants, which aimed to record the post-disaster evacuation behavior of the residents. The questionnaire was administered to all WBC participants during the waiting time for the screening, and they were self-completed and returned before starting the screening; the response rate was nearly 100%. In the case of children, the accompanying parents completed the questionnaire. This survey included questions on residents' evacuation behavior within the first month after the Fukushima disaster (i.e. March 11 to March 31, 2011), including whether they took part in evacuation(s), and the time-period and location of each evacuation. In addition, the survey collected household information, such as whether the participants were, at the time of the WBC screening, living with pre-school children; living with elderly people aged 70 years or older; or living alone.

The questionnaire on evacuation behavior was administered to those undertaking the screening program for the first time. In later rounds of WBC screening, the questionnaire was not administered. Note that when the WBC screening was launched in July 2011, appointments were booked more than half a year in advance. Due to the large number of pre-booked appointments, residents could usually not undergo screening more than once until March 2013. After that, it was possible for residents to undergo screening a second time.

### Data collection

We extracted data from the evacuation behavior survey for those who participated in the WBC screening between July 11, 2011 (inception of the screening and survey) and April 30, 2013. The data comprised of: sex; age at WBC screening; family identification number (given to all WBC participants to enable linkage to other family members' data); residential address before the disaster; evacuation behavior until March 30, 2011, including if, when, and to where the participants evacuated, and when and to where they returned; and household information at the time of WBC screening (described above). Information regarding participants’ addresses pre- and post-disaster were declassified at the lowest hierarchical level of administrative divisions of the city (i.e. O-aza, which is the sub-district). Note that there were 31, 63, and 41 O-azas in the mandatory evacuation zone, indoor sheltering zone, and non-evacuation zone respectively.

For baseline pre-disaster population demographics of Minamisoma City, we also used tabulated data on the population (organized according to sex, age, and address at the O-aza level) as of March 1, 2011, which were collected from the “Basic Resident Register”—a nationwide resident registry network, administrated by each municipality unit (city, town, or village). This register contains basic data about registered residents, including information regarding residents’ addresses. However, after the Fukushima disaster, evacuees often neglected to update their addresses recorded in the Basic Resident Register in the municipality near their original place of stay, and therefore the registered residential addresses post-disaster do not necessarily indicate their current address. For clarification, we hereafter use the term 'dwelling area' to refer to pre-disaster residential addresses as well as post-disaster current addresses that were obtained from the questionnaire on evacuation behavior.

### Data analysis

To estimate post-disaster demographic transitions in Minamisoma City during the first month after the disaster (from March 11 to March 31, 2011), and to identify the potential demographic factors associated with the population remaining in place, we performed the following three analyses:

#### Analysis 1: Modeled estimates of the population aged 6 years or older, during the first month of the disaster

We used the following model to estimate the total population in Minamisoma City at *k* days following the disaster:

$$P_{k}=\sum_{a=1}^{A}\sum_{b=1}^{B}\sum_{c=1}^{C}N_{a,b,c,k}\times \frac{1}{R_{a,b,c}}$$

where *N*_*a*,*b*,*c*,*k*_ is the number of individuals of sex *a*, in the age group *b*, at the dwelling area *c*, at *k* days following the disaster among WBC participants. *R*_*a*,*b*,*c*_ indicates the magnification coefficient for a group of sex *a*, in the age group *b*, at the dwelling area *c*, which was calculated using the following formula:

$$R_{a,b,c}=\frac{N_{a,b,c}^{\prime }}{P_{a,b,c}^{\prime }}$$

where $N_{a,b,c}^{\prime }$ is the whole number of individuals of sex *a*, in the age group *b*, at the dwelling area *c*, who had participated in WBC screening program during the study period (July 11, 2011–April 30, 2013). $P_{a,b,c}^{\prime }$ is the pre-disaster population as of March 1, 2011 of sex *a*, in the age group *b*, at the dwelling area *c*. We used data from the Basic Resident Register for $P_{a,b,c}^{\prime }$. Therefore, *R*_*a*,*b*,*c*_ refers to, in other words, the ratio of the number of WBC participants to the pre-disaster population of sex *a*, in the age group *b*, at the dwelling area *c*. For example, if all individuals of sex *a*, in the age group *b*, at the dwelling area *c* had participated in the WBC screening program during the study period, *R*_*a*,*b*,*c*_ equals to 1. Similarly, if no individuals had participated, *R*_*a*,*b*,*c*_ equals to 0.

To maximize the predictive power of this model, we compared the 'predictability' (defined below) of the following three models in relation to sex, age group, and dwelling area for the population estimates:

(I) Sex: male and female; age group: one-year age intervals; and dwelling area: mandatory evacuation zone, indoor sheltering zone, and other areas
(II) Sex: male and female; age group: five-year age intervals, and dwelling area: at O-aza level
(III) Sex: male and female; age group: 6–9, 10–14, 15–19, 20–39, 40–64, 65–74, and 75 and above (age in years), dwelling area: at O-aza level.

Using *P*′, the total number of pre-disaster population of both sexes, in all age groups, and at all dwelling areas, the 'predictability’ of each model was defined as follows:

$$Predictability=\frac{1}{P^{\prime }}\times \sum_{a=1}^{A}\sum_{b=1}^{B}\sum_{c=1}^{C}\{\begin{array}{l} P_{a,b,c}^{\prime }\;(N_{a,b,c}^{\prime }>0)\\ \;0\;(N_{a,b,c}^{\prime }=0) \end{array}$$

where the numerator indicates the modeled estimates of the total pre-disaster population in Minamisoma City as of March 1, 2011, while the denominator is the 'actual' total pre-disaster population in the city as of March 1, 2011. Therefore, the predictability can be regarded as the ratio of the modeled estimates to the actual pre-disaster population in the city. By default, if the WBC participants included both sexes, all age groups, and all dwelling areas, predictability can be calculated to be 100%. The model with the highest predictability among three models shown above (I, II, III) was adopted in this study as the best method for estimating the pre-disaster population of Minamisoma City.

#### Analysis 2: Modeled estimates of the population younger than 6 years of age, during the first month of the disaster

Because the WBC screening was conducted for residents aged 6 years or older, post-disaster evacuation behavior in children aged less than 6 years (i.e. pre-school children) was unknown. However, we assumed that evacuation behavior of pre-school children would be identical to that of their family members. Family identification numbers enabled linkage of the data concerning pre-school children to that of their family members (if they also participated in the WBC screening). Based on this assumption, we estimated the total population of residents aged less than 6 years in Minamisoma City at *k* days following the disaster, using the same model and model adopted in Analysis 1, and by employing a single age group (i.e. 0–5 years). We used the data on pre-school children when all other family members’ (e.g. parents) evacuation behavior was identical within that family.

#### Analysis 3: Factors associated with the population remaining in place

To investigate factors related to the population remaining in place after voluntary evacuation, multiple regression analysis was performed. For this analysis, we defined 'evacuee/evacuation' as those who evacuated to a location outside Minamisoma City once or more before March 31, 2011 (even if they had returned to an area inside the city, or to the original pre-disaster dwelling area, before this date), while 'remainee/remaining in place' was defined as evacuation within the city, or non-evacuation. To exclude the effect of mandatory evacuation on this analysis, we exclusively used data on those living in the indoor sheltering zone and other areas outside the mandatory evacuation zone at the time of the disaster, as indicated by the pre-disaster dwelling address (i.e. Basic Resident Register).

The probit regression model—a non-linear regression model where the dependent variable is a binary variable (i.e. evacuate or remain in place)—was employed in this study. We incorporated six independent variables into the regression model: one categorical variable (age—using age groups adopted in Analysis 1) and five binary values (sex–male or female; pre-disaster dwelling area–indoor sheltering zone or other areas of the city; at the time of WBC screening, living with pre-school children—yes or no; living with an elderly person aged 70 years or older—yes or no; and living alone—yes or no). For a detailed explanation of this approach, see online supplementary S1 Text.

### Ethics

The Institutional Review Board of Minamisoma Municipal General Hospital approved this study (authorization number 28–02). For the use of questionnaire data, written informed consent was obtained from the WBC participants, and the parents/guardians of participating children. For all analyses, we used R version 3.30. P values of less than 0.05 were considered statistically significant.

## Results

According to the Basic Resident Register, the total population in Minamisoma City as of March 1, 2011 was 70,919 and population aged 6 years or older was 67,929 (96%) (S1 Table). Among this population, 12,201 people lived in the mandatory evacuation zone before the disaster, 44,773 in the indoor sheltering zone, and 10,955 in other areas of the city, indicating that 28% (3,415/12,201), 31% (13,801/44,773), and 27% (2,933/10,955) of the residents in each of these areas respectively participated in the WBC screening at MMGH during the study period.

**Fig 2:**
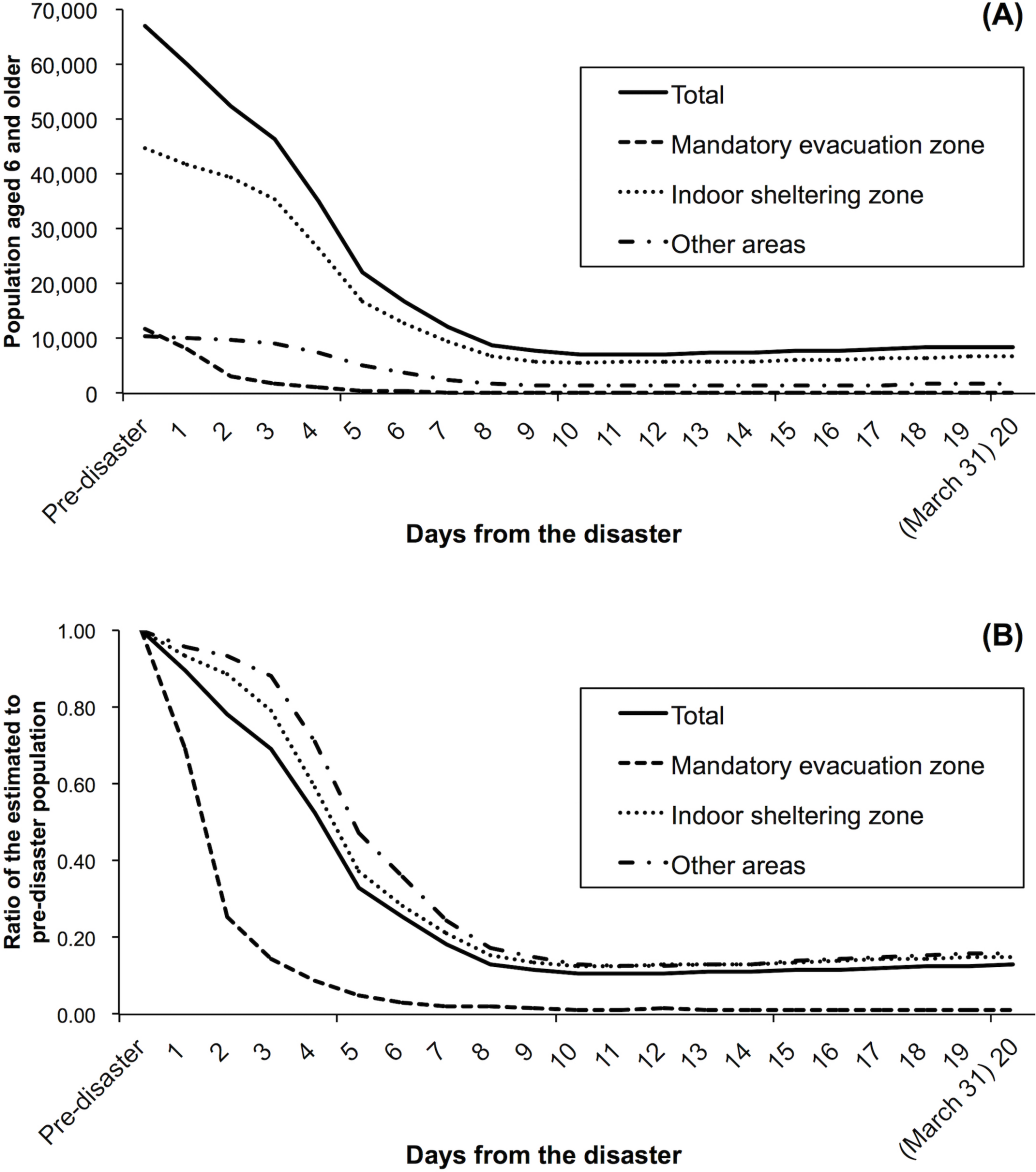
**Trends in (A) the modeled estimates of post-disaster population for those aged 6 years or older in Minamisoma City and (B) the ratio of estimated population to pre-disaster population in each of the three distinct zones. ‘**Pre-disaster’ corresponds to March 1, 2011. Day 4 (March 15) was the day when the indoor sheltering instruction was issued.

The population predictability for each model (I–III) was 96.3%, 96.9%, and 98.7%. Therefore, we adopted model III. The median of *R*_*a*,*b*,*c*_ in model III was 0.27 (0.00–1.00). Fig 2 illustrates the modeled estimates of the population aged 6 years or older in Minamisoma City between March 11 and 31, 2011. Actual values for the modeled estimates of this population during this period can be found in S2 Table. The pace of population decline was most striking in the first week of the disaster, but it substantially differed between different areas of the city. For instance, people in the mandatory evacuation zone were more likely to evacuate faster than those living in indoor sheltering zone/other areas, particularly in the first two days after the disaster. From March 15, 2011 (four days following the disaster), when the indoor sheltering instruction was issued by the central government, the pace of population decline became much faster in the indoor sheltering zone and other areas. The population decline in Minamisoma City peaked on March 22, 2011, 11 days following the disaster (n = 7,107, 11% of the pre-disaster level). On analysis of the population size in each area in the city, we found that the population sizes were at their lowest on March 22, 21, and 23, for the mandatory evacuation zone (132, 1.1%), indoor sheltering zone (5,595, 13%), and other areas of the city (1,333, 13%).

**Fig 3:**
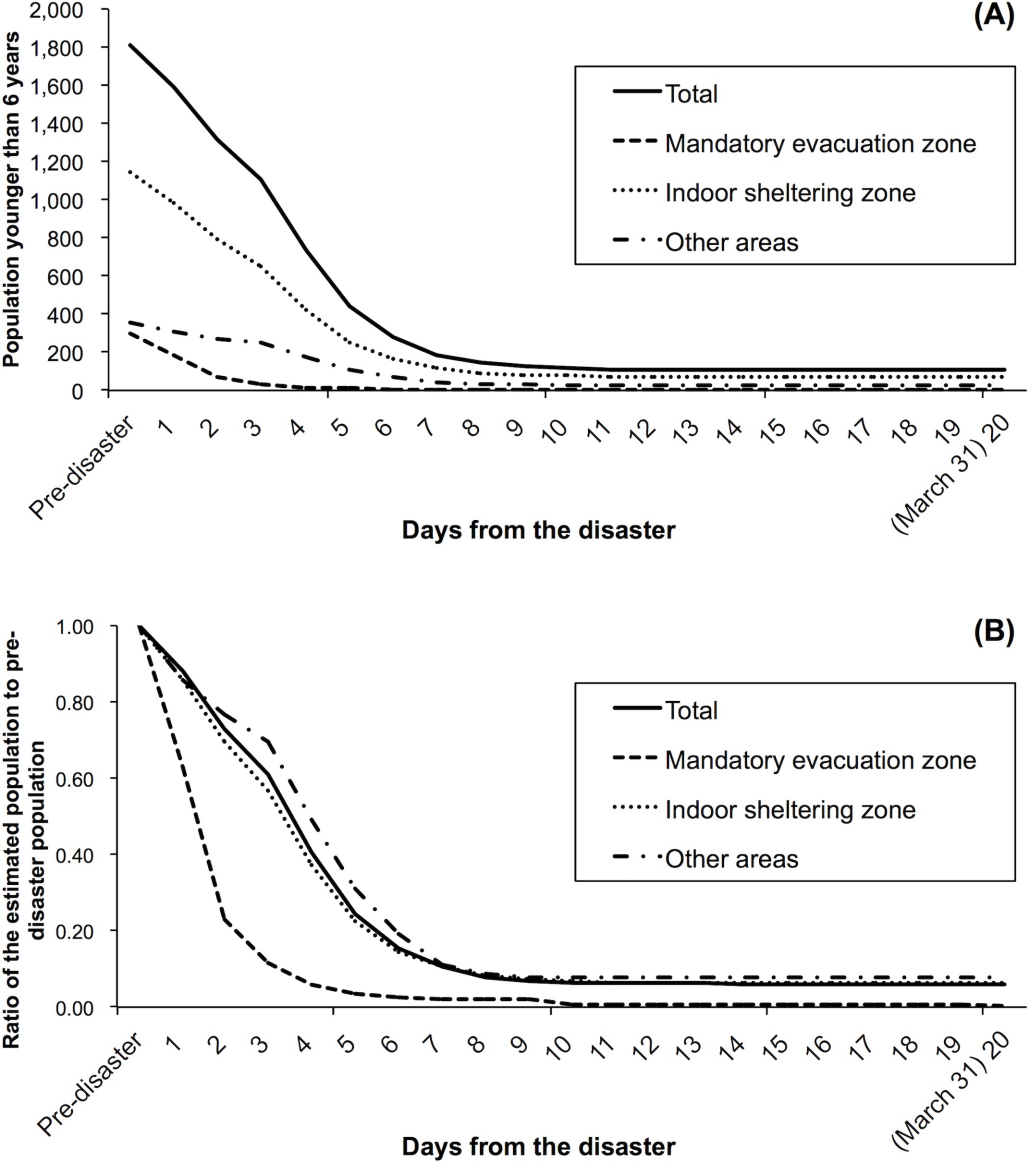
**Trends in (A) the modeled estimates of post-disaster population younger than 6 years in Minamisoma City and (B) the ratio of estimated population to pre-disaster population in each of the three distinct zones. ‘**Pre-disaster’ corresponds to March 1, 2011. Day 4 (March 15) was the day when the indoor sheltering instruction was issued.

Trends in the modeled estimates of the population of pre-school children (younger than 6 years old) in Minamisoma City between March 11 and 31, 2011 are shown in Fig 3. Actual values for the modeled estimates of the population can be found in S2 Table. In the mandatory evacuation zone, pre-school children also demonstrated a radical population decline in a similar manner to those aged over 6 years, with a peak on March 31, 2011 (1, 0.3%). On the other hand, in the indoor sheltering zone and other areas, the pace of the population decline among pre-school children, particularly in the first four days following the disaster (i.e. before the indoor sheltering instruction was issued), was much faster than among those aged over 6 years.

Using the data about residents who had lived in the indoor sheltering zone and other areas in Minamisoma City at the time of the disaster, we identified factors associated with the population remaining in place after the disaster (Table 2). After adjustment for covariates, women were less likely to remain in place during the first month after the disaster, compared to men, with an odds ratio (OR) of 0.58 (95% confidence intervals (CI): 0.54–0.61), p<0.001. In comparison with people in the indoor sheltering zone, those living in areas with no evacuation or indoor sheltering instructions were less likely to remain in place (OR: 0.80 [0.74–0.86], p<0.01). One to three-fold differences in odds for remaining in place were identified in the population, when classified according to age group: 6–9 years; 0.34 [0.27–0.42], p<0.001 (the lowest) and 40–64 years; 1.40 [1.24–1.58], p<0.01 (the highest), with reference to the age group of 75 years and above. Those living with pre-school children were more likely to evacuate (OR: 0.56 [0.50–0.62], p<0.001), while those living with elderly persons aged 70 years or older, and those living alone were more likely to remain in place (OR: 1.18 [1.03–1.34], p<0.05 and 1.71 [1.50–1.94], p<0.001, respectively).

**Table 2:** Odds ratios for remaining in Minamisoma City after the Fukushima disaster.

Variable	n	Odds ratio	95% CI	P-value
Sex				
Female	9,143	1.00		
Male	7,591	1.72	1.64–1.85	<0.001
Pre-disaster dwelling area				
Other areas	2,933	1.00		
Indoor sheltering zone	13,801	1.25	1.16–1.35	<0.01
Age at WBC screening [years]				
6–9	1,260	0.34	0.27–0.42	<0.001
10–14	2,117	0.37	0.31–0.44	<0.001
15–19	1,396	0.38	0.31–0.46	<0.001
20–39	3,123	1.18	1.03–1.34	0.22
40–64	5,654	1.40	1.24–1.58	<0.01
65–74	2,027	0.89	0.78–1.02	0.38
75–	1,157	1.00		
Household at WBC screening				
Living with pre-school children				
No	14,480	1.00		
Yes	2,254	0.56	0.50–0.62	<0.001
Living with the elderly aged 70 years or older				
No	12,233	1.00		
Yes	4,501	1.18	1.09–1.27	<0.05
Living alone				
No	16,114	1.00		
Yes	620	1.71	1.50–1.94	<0.001

CI: confidence interval. Data for those living in the mandatory evacuation zone at the time of the disaster were not included. Variables in the table are mutually adjusted.

## Discussion

This is the first study describing demographic changes as a result of evacuation in the acute phase of the 2011 Fukushima nuclear disaster. This study finds that the overall population in Minamisoma City decreased to 11% of the population before the disaster (7,107/67,044) on day 11 of the nuclear disaster (March 22, 2011). This result is comparable to a previous study on mobile phone services, in which the number of residents in Minamisoma City was estimated to have decreased to less than 10,000 within a month of the disaster [10, 11].

This study illuminates the fact that there was mass voluntary evacuation even outside of the mandatory evacuation zone. Nearly 90% of residents evacuated voluntarily from both the indoor sheltering zone and from other areas of the city. Voluntary evacuation after a nuclear disaster can be speculated to be caused by multiple reasons, including fear of a worsened disaster situation. Past reports suggest that many residents felt uncertainty about the truthfulness of information provided by the government after the nuclear disaster, which may have led to anxiety and overestimation of radiation exposure and related health impacts [16, 17]. Another possible reason for mass voluntary evacuation is shortage of resources. For example, a past report on the Fukushima disaster suggested that the staff of a hospital in the indoor sheltering zone chose to evacuate along with the patients, because the shortage of resources meant that they could not continue to provide services at the hospital [18].

Indoor sheltering instructions may have accelerated voluntary evacuation among residents in the indoor sheltering zone, and from other areas of the city. According to our results, the population of the indoor sheltering zone rapidly decreased after the indoor sheltering instructions were issued on March 15, 2011. The exact reason for this is unclear; however, indoor sheltering instructions may have provoked fear among residents, and also among people normally entering and leaving Minamisoma City for business. For example, some transport companies refused to let employees enter the indoor sheltering zone after the issue of indoor sheltering instructions, for fear of potential irradiation among employees, leading to exacerbation of shortages in resources, such as food and medical supplies [18]. Though indoor sheltering instructions were an effective countermeasure to prevent additional radiation exposure among residents [19], it is possible that they also led to disorganized mass voluntary evacuation after the Fukushima nuclear disaster. In previous disasters, disorganized evacuation was reported to increase morbidity and mortality rates in affected areas, due to loss of access to appropriate medical care and adequate supply of resources [6, 20]. Past reports by World Health Organization and United Nations Scientific Committee on the Effects of Atomic Radiation suggest that these evacuation orders were calculated to avert, at most, 50 mSV of radiation exposure [21, 22]. However, previous research also points out increased mortality and chronic disease prevalence due to secondary health effects of evacuation behaviors (e.g. community disruption) especially among the elderly [23, 24]. As a result, one report indicates that the health effects of radiation exposure were less serious than those related to the post-disaster increase in diabetes [25]. In addition, the timing of issues of evacuation instructions may be worth considering. The pace of population decline escalated after evacuation or sheltering instructions were issued, both in the mandatory evacuation zone and indoor sheltering zone. Because post-disaster situations vary greatly, it is difficult to generalize the optimal timing of evacuation orders. However, under circumstances where mandatory evacuation should be prioritized or supported, it could be appropriate to issue mandatory evacuation orders prior to other kind of evacuation-related instructions (e.g. indoor sheltering instructions). Policy makers should issue evacuation instructions at appropriate timings while being aware of the potential risks of evacuation instructions and with preparation to mitigate such risk [25].

Notably, this study suggests that there were remainees in the mandatory evacuation zone, even after mandatory evacuation instructions. In this zone, 99% (12,524/12,694) of the residents were estimated to have evacuated within ten days of the nuclear disaster. This finding is comparable with the situation after the 1987 Chernobyl nuclear disaster. Some residents, referred to as *samosely*, voluntarily lived in the exclusion zone surrounding the damaged Chernobyl nuclear power plant [26]. Because no residents were supposed to live in the Chernobyl mandatory evacuation zone, the remaining residents in the Chernobyl mandatory evacuation zone were likely unable to receive public support (e.g. medical insurance or pensions) [27]. In any case of future nuclear disasters, it is important to note that some residents in mandatory evacuation zones may remain in place despite evacuation orders, and to adequately prepare and respond to their needs.

These findings have some policy implications. First, disorganized evacuation after indoor-sheltering instructions, potentially amplifying the health effects seen in the indoor-sheltering zone and other areas of the city after the nuclear disaster, might be mitigated with political support. In the future, it may be helpful for indoor sheltering instructions to be accompanied with measures to manage fear and anxiety, through active disclosure of information on the dispersion of radioactive materials and air radiation doses [28, 29]. It may additionally be helpful to provide adequate support, including transportation or shelter, for residents in indoor-sheltering zone who hope to voluntarily evacuate, in order to prevent disorganized evacuations. Furthermore, it is important to note that issuing multiple types of evacuation instructions within a municipality could provoke fear and confusion. While a past study suggests that evacuation orders should be adjusted according to population distribution and contamination levels [30], in the case of the Fukushima disaster, it may have been more practical to issue evacuation instructions by municipality level rather than distance from the plant and ambient dose levels, in order to reduce the potential for fear, confusion, and disorganized evacuations that may have been prompted by multiple evacuation orders within the same municipality.

Overall, our findings suggest that voluntary evacuation behavior after the nuclear disaster had different patterns when assessed by sex, age group and household composition. The demographic factors associated with remaining in Minamisoma City were: being male, aged from 40 to 64 years, living with an elderly person, and living alone whereas those associated with voluntary evacuation from the city were; being female, younger than 20 years of age, and living with children. Differences in voluntary evacuation behavior may be related to known differences in radiation sensitivity depending on age: For example, children are more sensitive to radiation exposure than the elderly. Furthermore, psychological stress after nuclear disasters is reported to generally be greater among women than men [31], and a systematic review has reported that women are more likely to evacuate than men because of sex differences in social roles, evacuation incentives, exposure to risk, and perceived risk, which is in agreement with our findings [32]. Notably, residents living with an elderly person or those living alone in the indoor sheltering zone and other areas of the city tended to remain in place. While the exact reason for this is still unknown, it may be related to the impaired physical mobility of the elderly, or social and/or economic limitations such as lack of information or financial resources [10, 33]. Further research is needed to elucidate the reasons for the differences in evacuation behavior observed in this study.

### Strengths and limitations

This study assessed population changes in the mandatory evacuation zone, indoor sheltering zone, and other areas of Minamisoma City after the 2011 Fukushima nuclear disaster. This study has three strengths: First, it is based on the most participated screening program to date, which has yielded a considerable amount of information about the evacuation. Though a previous study assessed evacuation behavior after the nuclear disaster, based on mobile phone service users, the service users were limited to 0.7% of the whole population [13]; thus, the present study is likely to be more accurate, as it is based on data collected through the WBC screening, which covered approximately one-third of the population of Minamisoma City. Second, this study is the first to assess differences in evacuation behavior between the indoor sheltering zone and other areas of the city after the nuclear disaster. Finally, this study includes variables such as sex, age, and household composition, making it the first to investigate differences in evacuation behavior based on type of evacuation and demographic characteristics.

The limitation of this study is its potential for selection bias. The participants of this study attended the voluntary WBC screening program, and thus may have been more concerned about radiation exposure than other (non-participating) residents. It has been reported that those who are concerned about radiation exposure tend to have high evacuation rates [34]. Therefore, the participants in this study may have had a higher prevalence of evacuation than other residents, potentially contributing to an overestimation of the number of evacuees. It is additionally necessary to note that this study only considered the WBC screening conducted in Minamisoma City; we could not include residents who did not come back to the city after evacuation. Hence, it is also possible that we may have underestimated the number of evacuees. The combined effect of the above issues could have led to either overestimation or underestimation of the number of remainees in this study.

Moreover, the participation rate in the WBC screening differed by age. The participation rate was 71% (2,364/3,334) for those aged 10 to 14 years, whereas the participation rate was 15% (1,488/9,997) for those aged 75 years or older (Table 1; S1 Table). The population estimates in our study may therefore be more accurate for those with higher participation rates (i.e. the 10 to 14 year-old population). In addition to this selection bias, the possibility of recall bias is another limitation to the present study. As the information on evacuation behavior relies on the memory of the participants in the WBC screening, the recorded time of evacuation may be inaccurate [35].

## Conclusions

This study demonstrates changes in population demographics after the Fukushima Daiichi nuclear power plant disaster. There was mass, although not total, evacuation from the mandatory evacuation zone (99% of residents), and from the indoor sheltering zone and other areas of Minamisoma City (90% of residents) within the first month after the disaster. Despite evacuation orders, some residents of the mandatory evacuation zone remained in place. Inadequate indoor sheltering instructions may have contributed to disorganized voluntary evacuation. Further, voluntary evacuation behavior differed according to sex, age group and household composition. These findings may be of use to guide future dissemination of indoor sheltering or voluntary evacuation orders, focusing on minimizing disorganized evacuation in future nuclear events. Our findings may also help in anticipating which population groups (e.g. the elderly, men living alone) may remain in place, and require public assistance, such as medical care, in areas affected by nuclear disasters.

## Supporting information

S1 Table Demographic characteristics of the pre-disaster population (as of March 1, 2011). (DOCX)

S2 Table Modeled estimates of the population in Minamisoma City from March 1 to 31, 2011, by pre-disaster dwelling area. (DOCX)

S1 Fig Trends in the number of post-disaster population aged 6 and over estimated based on model (I) and model (II) in Minamisoma City. Gray lines show trends estimated based on model (III).
(TIFF)

S1 Text Probit regression model. (DOCX)
