# Supplementary material for: Demographic transition and factors associated with remaining in place after the 2011 Fukushima nuclear disaster and related evacuation orders
Source: PLoS One. 2018 Mar 14;13(3):e0194134. doi: 10.1371/journal.pone.0194134 (PMC5851610; doi:10.1371/journal.pone.0194134)
Supplement: S1 Table — (DOCX) [file pone.0194134.s001.docx]

**S1 Table. Demographic characteristics of the pre-disaster population (as of March 1, 2011)**

| Dwelling area | Mandatory evacuation zone  (n=12,694) | Indoor sheltering zone  (n=46,830) | | Other areas in the city  (n=11,395) | | Total  (whole areas)  (n=70,919) | |  |
| --- | --- | --- | --- | --- | --- | --- | --- | --- |
| Sex (n, %) |  |  | |  | |  | |  |
| Male | 6,104 (48) | 22,896 (49) | | 5,546 (49) | | 34,546 (49) | |  |
| Female | 6,590 (52) | 23,934 (51) | | 5,849 (51) | | 36,373 (51) | |  |
| Age group as of March 1, 2011 [years] | | |  | |  | |  | |
| 0–5 | 493 (4) | 2,057 (4) | | 440 (4) | | 2,990 (4) | |  |
| 6–9 | 622 (5) | 2,266 (5) | | 511 (4) | | 3,399 (5) | |  |
| 10–14 | 566 (4) | 2,239 (5) | | 529 (5) | | 3,334 (5) | |  |
| 15–19 | 670 (5) | 2,282 (5) | | 475 (4) | | 3,427 (5) | |  |
| 20–39 | 2,478 (20) | 10,465 (22) | | 2,345 (21) | | 15,288 (22) | |  |
| 40–64 | 4,384 (35) | 15,979 (34) | | 3,940 (35) | | 24,303 (34) | |  |
| 65–74 | 1,435 (11) | 5,442 (12) | | 1,304 (11) | | 8,181 (12) | |  |
| 75– | 2,046 (16) | 6,100 (13) | | 1,851 (16) | | 9,997 (14) | |  |
