# Supplementary material for: Demographic transition and factors associated with remaining in place after the 2011 Fukushima nuclear disaster and related evacuation orders
Source: PLoS One. 2018 Mar 14;13(3):e0194134. doi: 10.1371/journal.pone.0194134 (PMC5851610; doi:10.1371/journal.pone.0194134)
Supplement: S2 Table — (DOCX) [file pone.0194134.s002.docx]

**S2 Table. Modeled estimates of the population in Minamisoma City from March 1 to 31, 2011, by pre-disaster dwelling area**

|  | Pre-disaster |  | Days from the Fukushima disaster (e.g. 1; March 12, 2011, 20; March 31, 2011) | | | | | | | | | | | | | | | | | | | |
| --- | --- | --- | --- | --- | --- | --- | --- | --- | --- | --- | --- | --- | --- | --- | --- | --- | --- | --- | --- | --- | --- | --- |
|  | (March 1, 2011) |  | 1 | 2 | 3 | 4 | 5 | 6 | 7 | 8 | 9 | 10 | 11 | 12 | 13 | 14 | 15 | 16 | 17 | 18 | 19 | 20 |
| Total |  |  |  |  |  |  |  |  |  |  |  |  |  |  |  |  |  |  |  |  |  |  |
| Population | 70,919 |  | 63,548 | 55,562 | 49,051 | 37,067 | 23,522 | 17,840 | 12,934 | 9,388 | 8,151 | 7,550 | 7,518 | 7,631 | 7,731 | 7,909 | 8,067 | 8,265 | 8,569 | 8,770 | 8,945 | 9,093 |
| (%) | (100) |  | (90) | (78) | (69) | (52) | (33) | (25) | (18) | (13) | (11) | (11) | (11) | (11) | (11) | (11) | (11) | (12) | (12) | (12) | (13) | (13) |
| Younger than 6 | 2,990 |  | 2,628 | 2,176 | 1,822 | 1,214 | 726 | 455 | 313 | 235 | 207 | 192 | 184 | 184 | 184 | 182 | 182 | 180 | 180 | 179 | 179 | 179 |
| (%) | (100) |  | (88) | (73) | (61) | (41) | (24) | (15) | (10) | (8) | (7) | (6) | (6) | (6) | (6) | (6) | (6) | (6) | (6) | (6) | (6) | (6) |
| Mandatory evacuation zone |  |  |  |  |  |  |  |  |  |  |  |  |  |  |  |  |  |  |  |  |  |  |
| Population | 12,694 |  | 8,788 | 3,222 | 1,833 | 1,133 | 613 | 375 | 290 | 243 | 220 | 170 | 141 | 176 | 160 | 173 | 148 | 168 | 151 | 151 | 145 | 167 |
| (%) | (100) |  | (69) | (25) | (14) | (9) | (5) | (3) | (2) | (2) | (2) | (1) | (1) | (1) | (1) | (1) | (1) | (1) | (1) | (1) | (1) | (1) |
| Younger than 6 | 493 |  | 310 | 114 | 58 | 28 | 18 | 12 | 10 | 10 | 10 | 3 | 3 | 3 | 3 | 3 | 3 | 3 | 3 | 3 | 3 | 2 |
| (%) | (100) |  | (63) | (23) | (12) | (6) | (4) | (2) | (2) | (2) | (2) | (1) | (1) | (1) | (1) | (1) | (1) | (1) | (1) | (1) | (1) | (0) |
| Indoor evacuation zone |  |  |  |  |  |  |  |  |  |  |  |  |  |  |  |  |  |  |  |  |  |  |
| Population | 46,830 |  | 43,783 | 41,519 | 37,065 | 27,743 | 17,478 | 13,317 | 9,816 | 7,136 | 6,217 | 5,873 | 5,912 | 5,990 | 6,076 | 6,204 | 6,314 | 6,436 | 6,663 | 6,818 | 6,980 | 7,078 |
| (%) | (100) |  | (93) | (89) | (79) | (59) | (37) | (28) | (21) | (15) | (13) | (13) | (13) | (13) | (13) | (13) | (13) | (14) | (14) | (15) | (15) | (15) |
| Younger than 6 | 2,057 |  | 1,765 | 1,431 | 1,165 | 766 | 460 | 297 | 215 | 166 | 148 | 139 | 132 | 132 | 132 | 130 | 130 | 128 | 128 | 128 | 128 | 128 |
| (%) | (100) |  | (86) | (70) | (57) | (37) | (22) | (14) | (10) | (8) | (7) | (7) | (6) | (6) | (6) | (6) | (6) | (6) | (6) | (6) | (6) | (6) |
| Other areas in the city |  |  |  |  |  |  |  |  |  |  |  |  |  |  |  |  |  |  |  |  |  |  |
| Population | 11,395 |  | 10,890 | 10,655 | 10,015 | 8,110 | 5,373 | 4,094 | 2,777 | 1,983 | 1,692 | 1,485 | 1,442 | 1,441 | 1,472 | 1,509 | 1,581 | 1,633 | 1,728 | 1,772 | 1,791 | 1,821 |
| (%) | (100) |  | (96) | (94) | (88) | (71) | (47) | (36) | (24) | (17) | (15) | (13) | (13) | (13) | (13) | (13) | (14) | (14) | (15) | (16) | (16) | (16) |
| Younger than 6 | 440 |  | 377 | 337 | 305 | 216 | 136 | 85 | 49 | 38 | 35 | 33 | 33 | 33 | 33 | 33 | 33 | 33 | 33 | 33 | 33 | 33 |
| (%) | (100) |  | (86) | (77) | (69) | (49) | (31) | (19) | (11) | (9) | (8) | (8) | (8) | (8) | (8) | (8) | (8) | (8) | (8) | (8) | (8) | (8) |
