# Supplementary figures and images for: Demographic transition and factors associated with remaining in place after the 2011 Fukushima nuclear disaster and related evacuation orders
Source: PLoS One. 2018 Mar 14;13(3):e0194134. doi: 10.1371/journal.pone.0194134 (PMC5851610; doi:10.1371/journal.pone.0194134)

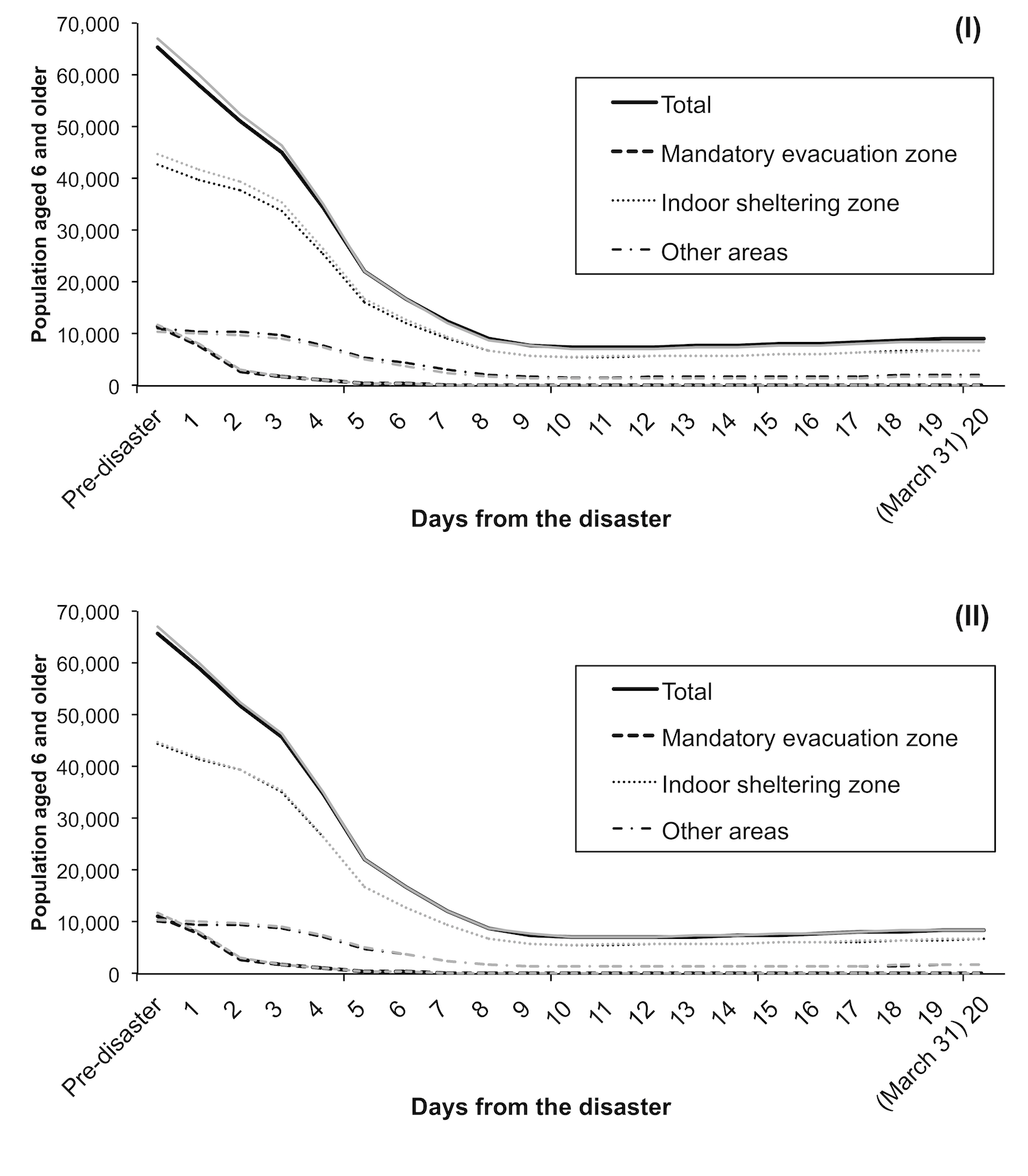

Supplement: S1 Fig — Gray lines show trends estimated based on model (III). (TIFF) [file pone.0194134.s003.tiff]
