# Supplementary material for: Demographic transition and factors associated with remaining in place after the 2011 Fukushima nuclear disaster and related evacuation orders
Source: PLoS One. 2018 Mar 14;13(3):e0194134. doi: 10.1371/journal.pone.0194134 (PMC5851610; doi:10.1371/journal.pone.0194134)
Supplement: S1 Text — (DOCX) [file pone.0194134.s004.docx]

**S1 Text. Probit regression model**

Probit regression models are employed in this study as the dependent variable is a binary variable (0: evacuate and 1: remain in place).

We aimed to estimate the model as follows:

$$y^{*} = \beta_{1}x_{1} + \beta_{2}x_{2} + \beta_{3}x_{3} + \beta_{4}x_{4} + \beta_{5}x_{5} + \beta_{6}x_{6}+ \varepsilon$$

$$y=\left\{ \begin{aligned} 1, y^{*} >0 \\ 0, y^{*} \leq0 \end{aligned} \right.$$

We incorporated six independent variables into the models: one categorical variable ($x_{1}$ (age): 6–9, 10–14, 15–19, 20–39, 40–64, 65–74, and 75–) and five binary values ($x_{2}$ (sex): male or female; $x_{3}$ (pre-disaster dwelling area): indoor sheltering zone or other areas of the city; $x_{4}$ (living with pre-school children): yes or no; $x_{5}$ (living with an elderly person aged 70 years or older): yes or no; $x_{6}$ (living alone): yes or no.

As we assume that the unobservable term $\varepsilon$ is normally distributed, the probability of observing remaining in Minamisoma City ($y=1$) and evacuating from Minamisoma City ($y=0$) is given as:

$\Pr\left( y=1 | x_{1}, x_{2}, x_{3}, x_{4}, x_{5},x_{6} \right)=\Phi\left( \beta_{1}x_{1} + \beta_{2}x_{2} + \beta_{3}x_{3} + \beta_{4}x_{4} + \beta_{5}x_{5} + \beta_{6}x_{6} \right)$

$\Pr\left( y=0 | x_{1}, x_{2}, x_{3}, x_{4}, x_{5},x_{6} \right)=1-\Phi\left( \beta_{1}x_{1} + \beta_{2}x_{2} + \beta_{3}x_{3} + \beta_{4}x_{4} + \beta_{5}x_{5} + \beta_{6}x_{6} \right)$

where $\Phi()$ is the cumulative normal density function.
